# Recovery After Transcervical Fibroid Ablation Versus Minimally Invasive Myomectomy for Symptomatic Uterine Fibroids: A Randomised Controlled Trial

**DOI:** 10.1111/1471-0528.70081

**Published:** 2025-11-10

**Authors:** Felix Neis, Bernhard Kraemer, Armin Bauer, Tjeerd Dijkstra, Sabine Matovina, Annika Rohner, Sara Y. Brucker

**Affiliations:** ^1^ Department of Obstetrics and Gynecology University Hospital Tübingen Tübingen Germany

**Keywords:** myomectomy, TFA, transcervical fibroid ablation, uterine fibroid

## Abstract

**Objective:**

To evaluate early recovery outcomes with transcervical fibroid ablation (TFA) compared to minimally invasive myomectomy (MIM) in women with symptomatic uterine fibroids.

**Design:**

Randomised controlled trial.

**Setting:**

Tübingen University Hospital (Tübingen, Germany).

**Sample:**

Premenopausal women aged 18–50 years with symptomatic uterine fibroids.

**Methods:**

Participants were randomised to undergo TFA or MIM. The MIM group underwent laparoscopic myomectomy with concurrent hysteroscopic myomectomy if submucosal fibroids were present.

**Main Outcome Measures:**

The primary endpoint was the time to return to normal activities. Secondary outcomes included procedure time, postprocedural pain, hospital discharge readiness, time to return to 10 additional activities of daily living, and adverse events. Clinical outcomes through 7 weeks of follow‐up were reported. The primary endpoint was evaluated at *p* < 0.028 due to a pre‐planned interim analysis; secondary outcomes were evaluated at *p* < 0.05.

**Results:**

Among 144 randomised patients, 119 provided follow‐up data (58 TFA; 61 MIM). The primary endpoint was met with the median time to return to normal activities favouring TFA (5.5 vs. 13 days; log‐rank *p* < 0.001). Procedure time (51 ± 21 vs. 95 ± 37 min; *p* < 0.001), postprocedural pain through discharge (all *p* < 0.01), opioid utilisation (25.9% vs. 49.2%, *p* = 0.009), and time to discharge readiness (22.9 ± 13.2 vs. 58.9 ± 33.1 h; *p* < 0.001) favoured TFA. Nine of 10 treatment recovery metrics statistically favoured TFA with none favouring MIM. One serious adverse event occurred in a patient treated with MIM (diagnostic laparoscopy for postoperative bleeding).

**Conclusions:**

TFA offers a faster recovery than MIM for the treatment of symptomatic uterine fibroids, with a comparable short‐term safety profile.

**Trial Registration:**

This trial was prospectively registered on the German Clinical Trials Register; https://drks.de/search/de/trial/DRKS00028847

## 1 Introduction

Uterine fibroids are the most common benign gynecologic tumours in women, affecting up to 70%–80% of women by age 50 [1, 2]. Up to half of women with fibroids experience symptoms that interfere with their daily activities, sexual function, work performance, and personal relationships [3, 4, 5]. Treatment selection is influenced by symptom severity, treatment risks, desire for uterus preservation, and future fertility considerations. Although medical therapy represents an important treatment option, it may not be suitable or effective for all patients [6]. Consequently, hysterectomy is often recommended as the only definitive treatment [7]. However, concerns about hysterectomy overutilization have emerged [8] as the procedure prevents future pregnancy, carries operative risks, and may lead to earlier menopause with associated health consequences [6, 9, 10].

Most women with symptomatic fibroids prefer uterus‐conserving treatments irrespective of their childbearing intentions [6]. Myomectomy is a standard treatment for women seeking symptom relief and fertility preservation, but carries risks of surgical complications and adhesion formation, and potential for fibroid recurrence [11, 12]. In contrast to transperitoneal myomectomy and hysterectomy, transcervical fibroid ablation (TFA) is an incisionless alternative that uses radiofrequency energy to ablate fibroids using real‐time intrauterine sonographic guidance [13, 14, 15, 16, 17]. While TFA has demonstrated favourable procedural safety and effectiveness with low reintervention rates [13, 14, 15, 16, 18], comparative evidence against other fibroid treatments is limited to nonrandomized studies [19, 20]. While limited data on pregnancy outcomes for TFA are available, current data demonstrate comparable live birth rates with ablative techniques and MIM [21, 22, 23].

Because functional recovery is a primary concern for many women considering uterine‐sparing treatment options, the SUPERIOR randomised controlled trial was designed to test the hypothesis that return to normal activity (RTNA) would be faster after TFA compared with minimally invasive myomectomy (MIM) in women with symptomatic uterine fibroids. This paper presents early recovery outcomes from the SUPERIOR trial.

## 2 Methods

### 2.1 Trial Design

We conducted a single‐center randomised controlled trial comparing TFA and MIM for uterine fibroid treatment at Tübingen University Hospital (Tübingen, Germany). The trial was designed with a 1‐year follow‐up period. This paper reports early (7‐week) recovery outcomes in study participants. The study protocol underwent review and approval by the institutional ethics committee and was prospectively registered on the German Clinical Trials Register (https://drks.de/search/de/trial/DRKS00028847). All participants provided written informed consent before enrollment. The study design, conduct, and reporting adhered to the Consolidated Standards of Reporting Trials (CONSORT) guidelines for randomised controlled trials [24].

### 2.2 Participants

Eligible participants were premenopausal women aged 18–50 years with symptomatic uterine fibroids of FIGO type 1, 2, 3, 4, 5, 6, or 2–5 [25] who provided informed consent and were seeking a uterus‐preserving fibroid treatment and for whom either TFA or MIM was equally considered an appropriate treatment option. Eligible participants had no more than 10 non‐pedunculated fibroids, each less than 8 cm in diameter, as determined by transvaginal ultrasound imaging. Exclusion criteria were clinically suggested severe endometriosis, anovulation, adenomyosis, immunocompromised state, pregnancy or breastfeeding, or contraindications to either procedure. Women who desired to preserve fertility for future pregnancy were counselled before enrollment on pregnancy outcomes after each technique based on available literature [23, 26].

### 2.3 Randomization and Blinding

A statistician who was independent of subject recruitment and other study procedures generated a simple 1:1 randomization sequence prior to participant enrollment to ensure allocation concealment. Treatment group assignments were placed in opaque, sequentially numbered envelopes sealed with adhesive closures. Each envelope was stamped across the seal to make it tamper‐evident and stored in a secure location accessible only to authorised personnel. After a participant provided written informed consent, the next envelope in sequence was opened to reveal the group assignment. Participants were blinded to their allocation until the procedure was completed.

### 2.4 Procedures

TFA was performed using the Sonata system (Gynesonics Inc., Redwood City, CA, United States), which integrates intrauterine ultrasound imaging with radiofrequency ablation for real‐time targeting and treatment of fibroids. The system consists of an integrated intrauterine sonography probe for visualisation and a radiofrequency handpiece for ablation. A graphical interface superimposed on the live ultrasound image delineated the target ablation area and extent of subablative thermal heating. This information enables the gynaecologist to confirm the ablation location within the fibroid while ensuring the thermal safety border remains within the uterine serosa. The TFA procedure has been described in detail elsewhere [17].

The MIM group underwent laparoscopic myomectomy, with concurrent hysteroscopic myomectomy if submucosal fibroids were present. MIM was performed using CO_2_ insufflation to establish pneumoperitoneum, allowing insertion of laparoscopic instruments through small incisions (typically 5–10 mm) in the umbilicus and lower abdomen (3–4 trocars). Fibroids were excised through hysterotomy incisions, and larger specimens were power morcellated for removal. In cases where submucosal fibroids were identified, hysteroscopic myomectomy was concurrently performed by employing a hysteroscope and bipolar electrosurgical loop to resect intramural fibroid tissue under saline solution distension.

Study procedures were performed by two experienced surgeons, each with previous experience in more than 100 minimally invasive, uterus‐sparing fibroid surgeries. Patients received general anaesthesia, and periprocedural care and analgesia protocols adhered to local institutional standards. In MIM cases with fibroid diameter less than 5 cm, day surgery was offered, while patients with fibroid diameter greater than 5 cm received inpatient care. Due to reimbursement policies in Germany, patients in the TFA arm received inpatient care regardless of fibroid size.

### 2.5 Postprocedural Recovery and Follow‐Up

Postprocedural vital signs were continuously monitored and recorded every 8 h until discharge. Inpatient management followed institutional and national guidelines. Discharge readiness was determined by clinical evaluation, stable vital signs, ability to ambulate, adequate pain control, absence of complications, and the patient's stated readiness for discharge. This approach enabled the assessment of postprocedural recovery independent of local institutional policies that influenced actual discharge timing.

Patients completed a treatment recovery questionnaire daily through the 48‐day follow‐up visit or until they reported returning to all specified activities. To ensure a high response rate to the questionnaires and record any adverse events during the follow‐up period, each patient was contacted by telephone after 30 and 45 days. Because published literature indicates that RTNA after MIM is generally longer than after TFA, the follow‐up period was based on typical recovery times for MIM (2–4 weeks) [27, 28]. To ensure complete assessment of RTNA in all participants, the follow‐up period was extended to 48 days.

### 2.6 Outcomes

The primary endpoint was the number of days from the procedure to RTNA, which was self‐reported in daily diaries and defined as the first post‐treatment day on which the patient reported returning to normal activities. Secondary outcomes included procedure time, postprocedural pain (reported on a 0–10 visual analogue scale), hospital discharge readiness, time to return to 10 additional activities of daily living reported on the treatment recovery questionnaire (regular diet, household tasks, lifting/carrying items, driving, recreational tasks, return to work, normal sleep, urination, bowel movements, sexual intercourse), adverse events, and surgical reinterventions.

### 2.7 Statistical Analysis

A sample size of 60 patients per group provided 93.5% power to detect a clinically meaningful difference of a mean of 2 days with a standard deviation of 3 days for RTNA between the TFA and MIM groups. A 2‐day difference in RTNA between groups assumed that a 1‐day earlier recovery would be clinically meaningful to patients, with an additional day added to account for variation in within‐day treatment timing; the 3‐day standard deviation represented moderate variability around that difference. The power analysis incorporated one pre‐planned interim analysis after primary endpoint data were collected for 30 patients per group, with the overall alpha level adjusted using the Pocock spending function [29]. Time to event outcomes were compared between groups using the log‐rank test. Additional outcomes were analysed using *t*‐tests for normally distributed continuous variables, Mann–Whitney *U* tests for non‐normally distributed continuous variables, and chi‐square tests for categorical variables. All analyses were performed using IBM SPSS Statistics v30.0 (IBM, Armonk, New York, United States). Kaplan–Meier curves were generated in R v4.3 (R Foundation for Statistical Computing, Vienna, Austria) using the *survival* package v3.8–3 and *ggsurvfit* v.1.1.0. The primary endpoint was assessed using a *p*‐value threshold of 0.028 for statistical significance. Additional outcomes were considered statistically significant at *p* < 0.05 without adjustment for multiplicity.

## 3 Results

**FIGURE 1:**
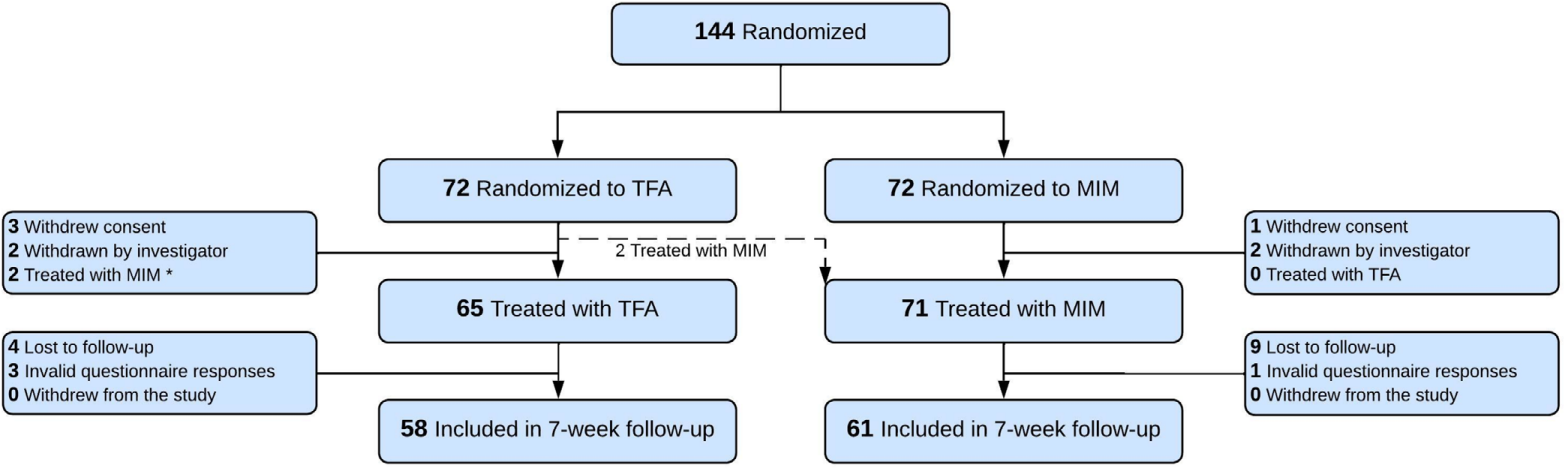
CONSORT flow diagram. MIM, minimally invasive myomectomy; TFA, transcervical fibroid ablation. *Two patients randomised to TFA were treated with MIM due to anatomical findings precluding TFA.

Between June 2022 and April 2025, 144 patients were randomised to receive TFA (*n* = 72) or MIM (*n* = 72), with 136 patients (65 TFA; 71 MIM) ultimately treated. Eight randomised patients were excluded prior to treatment due to withdrawn consent (4) or intraoperative findings indicating ineligibility for study participation (4), including fibroid diameter > 8 cm, the presence of more than 10 fibroids, severe endometriosis, and structural fibroid change with a sonographically suspicious ovary suggestive of concomitant pathology. Two patients assigned to TFA were found to be anatomically unsuitable for the procedure and instead underwent MIM. During follow‐up, 17 additional patients were excluded due to non‐response following multiple contact attempts for questionnaire completion or incomplete questionnaires that were missing the primary endpoint and other responses. Ultimately, 119 patients (58 TFA; 61 MIM) provided 7‐week follow‐up data (Figure 1).

Comparing patient characteristics in the TFA and MIM groups, the mean patient age was 38.6 ± 6.4 vs. 36.4 ± 5.5 years, the mean number of preprocedural identified fibroids per patient was 2.0 ± 1.4 vs. 1.8 ± 1.2, and the mean fibroid diameter was 3.9 ± 1.9 vs. 3.9 ± 2.0 cm, respectively (Table S1).

Concomitant procedures were performed more frequently in the MIM group (49.2% vs. 20.7%; *p* < 0.001) (Table 1). In the MIM group, 21 (34.4%) patients were treated for endometriosis, 5 (8.2%) received simultaneous polypectomy, 3 (4.9%) were treated with adhesiolysis, 2 (3.3%) received treatment for an ovarian cyst, and 1 (1.6%) underwent placenta remnant removal. Additionally, 14 (23.0%) were simultaneously treated with hysteroscopic myomectomy for submucosal fibroids. In the TFA arm, 8 (13.8%) patients underwent simultaneous polypectomy and 5 (8.6%) presented with submucosal fibroids deemed unsuitable for TFA and removed by curettage or hysteroscopy. Procedure time was significantly shorter with TFA compared to MIM (51 ± 21 vs. 95 ± 37 min; *p* < 0.001). The mean number of treated fibroids was 2.5 ± 1.9 with TFA and 2.7 ± 1.9 with MIM (*p* = 0.51), while the mean diameter of treated fibroids was larger in the TFA group (3.7 ± 2.0 vs. 3.2 ± 2.2 cm; *p* = 0.02) (Figure S1).

**TABLE 1:** Procedural characteristics with transcervical fibroid ablation (TFA) or minimally invasive myomectomy (MIM).

Variable^a^	TFA	MIM	*p*
No. patients (as treated)	58	61	
No. treated fibroids	136	160	
Mean treated fibroids per patient	2.5 ± 1.9 (1, 8)	2.7 ± 1.9 (1, 10)	0.51
Procedure time, min	51 ± 21 (18, 134)	95 ± 37 (25, 238)	< 0.001
Treated fibroid diameter, cm	3.7 ± 2.0 (0.8, 8.0)	3.2 ± 2.2 (0.3, 8.4)	0.02
Fibroid location relative to uterine wall			
Transmural	58 (42.6%)	48 (30.0%)	< 0.001
Intramural	51 (37.5%)	31 (19.4%)	
Subserosal	12 (8.8%)	54 (33.8%)	
Submucosal	15 (11.0%)	26 (16.3%)	
Other	0 (0%)	1 (0.6%)	
Fibroid location relative to uterine landmark			
Body	87 (63.9%)	105 (65.6%)	0.46
Fundal	44 (32.4%)	47 (29.4%)	
Lower segment	5 (3.7%)	7 (4.4%)	
Vaginal (paracervical)	0 (0%)	1 (0.6%)	
Combined hysteroscopy/laparoscopy	—	14 (23.0%)	
Conversion to laparotomy	—	1 (1.6%)	
No. untreated fibroids^b^	12 (8.1%)	1 (0.6%)	
Due to position	11 (7.4%)	1 (0.6%)	< 0.001
Due to size	1 (0.7%)	0 (0%)	
Patients with additional procedures^c^	12 (20.7%)	30 (49.2%)	
Endometriosis surgery	0 (0%)	21 (34.4%)	< 0.001
Peritoneal	0 (0%)	11 (18.0%)	
Deep	0 (0%)	10 (16.4%)	
Polypectomy	8 (13.8%)	5 (8.2%)	
Fibroid resection	5 (8.6%)^d^	0 (0%)	
Adhesiolysis	0 (0%)	3 (4.9%)	
Ovarian cyst	0 (0%)	2 (3.3%)	
Placenta remnant	0 (0%)	1 (1.6%)	

^a^ Values reported as count, mean ± SD (min, max), or *n* (%).

^b^ Percentages calculated where the denominator was the sum of treated and untreated fibroids identified during the procedure (148 with TFA; 161 with MIM).

^c^ The number of procedures exceeds the number of patients owing to multiple procedures in some individuals.

^d^ Resection of submucosal fibroids deemed unsuitable for TFA.

**FIGURE 2:**
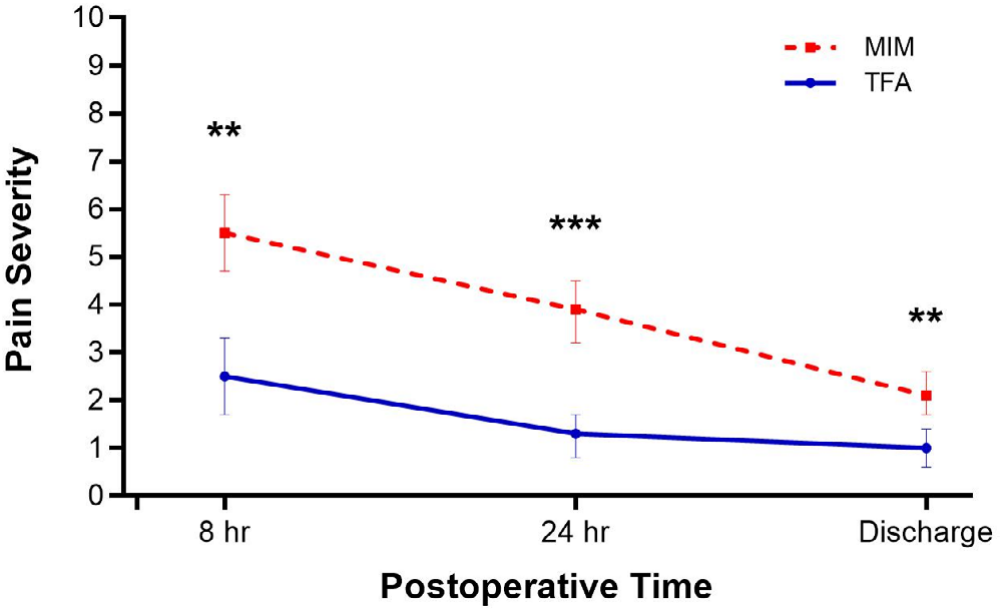
Comparison of postprocedural pain after transcervical fibroid ablation (TFA) and minimally invasive myomectomy (MIM). Pain severity reported on a 0–10 visual analogue scale. Error bars indicate 95% confidence intervals. ***p < 0.001; **p < 0.01.

Patients treated with TFA experienced less postprocedural pain at all measured time points through hospital discharge (all *p* < 0.01) (Figure 2). Postprocedural opioid utilisation was also lower after TFA vs. MIM (25.9% vs. 49.2%, *p* = 0.009), with median durations of use of 1 and 2 days, respectively. The mean time to discharge readiness was significantly shorter with TFA (22.9 ± 13.2 vs. 58.9 ± 33.1 h; *p* < 0.001).

**FIGURE 3:**
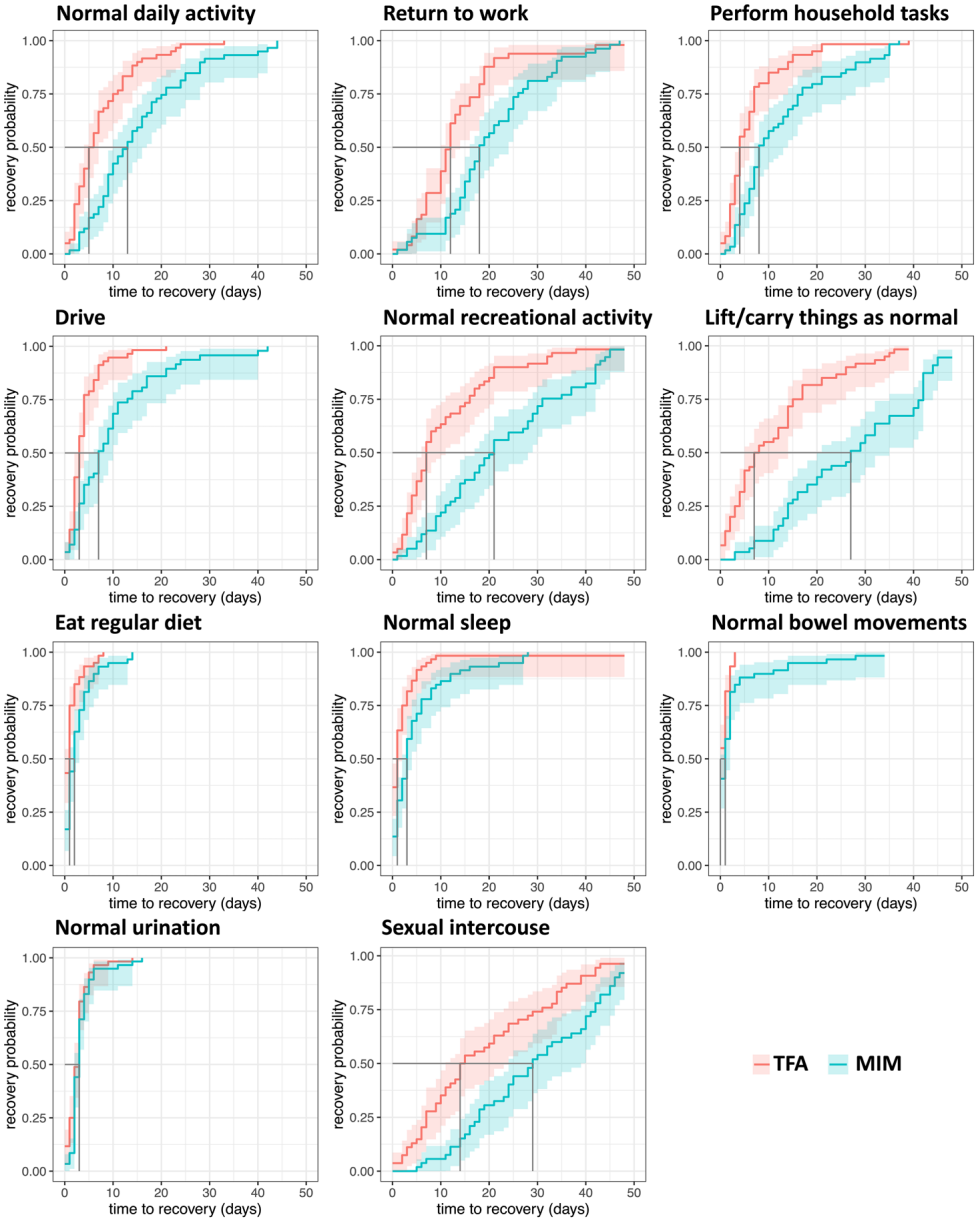
Comparison of time to return to activities after transcervical fibroid ablation (TFA) and minimally invasive myomectomy (MIM). Time to return to normal daily activity (top left panel) represents the primary endpoint of the trial. For this outcome, statistical significance was defined as *p* < 0.028 based on the interim analysis using Pocock's alpha spending function. The primary endpoint was achieved at *p* < 0.001. Secondary outcomes were considered statistically significant at *p* < 0.05. All treatment recovery outcomes statistically favored TFA, except for normal bowel movements which were not statistically different between groups. Shaded areas indicate 95% confidence intervals. Summary statistics and log‐rank *p*‐values are reported in Table S2.

The primary endpoint of time to RTNA was met, with TFA resulting in a shorter median recovery compared to MIM (5.5 vs. 13 days; *p* < 0.001). This *p*‐value was below the pre‐specified Pocock alpha boundary of 0.028. Of the 10 additional treatment recovery metrics assessed on the questionnaire, nine (household tasks, lifting/carrying items, driving, recreational tasks, return to work, urination, regular diet, normal sleep, and sexual intercourse) statistically favoured TFA, one (normal bowel movements) favoured neither group, and none favoured MIM (Figure 3, Table S2).

One serious adverse event was reported in the trial. A patient in the MIM group underwent diagnostic laparoscopy for postoperative bleeding 1 day after surgery (estimated blood loss 600 mL; haemoglobin decreased from 11.2 to 5.6 g/dL). No active bleeding source was identified; the patient declined transfusion and received 1 g of intravenous ferric carboxymaltose. Nonserious adverse events occurred in 9 (15.5%) patients in the TFA group and 3 (4.9%) patients in the MIM group (*p* = 0.08). The AEs reported in the TFA group included lower abdominal pain (5) vaginal bleeding (4), dizziness (2), and 1 case each of circulatory collapse, headache, nausea, urinary tract infection, and lower abdominal pain, and sleep disturbance. The AEs reported in the MIM group included vaginal bleeding (2) and one case each of lower abdominal pain, dizziness, and urinary tract infection. All adverse events occurred during the first 40 days after treatment and resolved completely within 2 weeks of onset. Aside from the previously described diagnostic laparoscopy, no surgical reinterventions were performed in either group during follow‐up.

## 4 Discussion

### 4.1 Main Findings

The results of this randomised controlled trial demonstrate that TFA enables a significantly faster RTNA compared to MIM in women with symptomatic uterine fibroids. Additionally, TFA resulted in shorter procedure times, reduced postprocedural pain, lower opioid utilisation, faster discharge readiness, and faster return to most daily activities assessed in the study. These outcomes are consistent with the transcervical route of TFA and suggest benefits in resource utilisation and patient recovery across multiple domains.

The procedure time for TFA was approximately half that of MIM, potentially allowing the treatment of more patients earlier and more effective utilisation of scarce operating room resources. The reduced frequency and duration of opioid use allow patients treated with TFA to be discharged in less than half the time of MIM. Furthermore, the shorter time to return to activities, including work, observed with TFA suggests potential indirect cost savings through reduced productivity loss. While this study did not directly assess economic outcomes, these results suggest that TFA may benefit both patient recovery and resource utilisation, warranting further investigation into its cost‐effectiveness compared to MIM.

### 4.2 Interpretation

The findings of this trial have important implications for clinical practice. Approximately one‐third of women with fibroids seek treatment, and often after considerable delay [6]. This may be partly due to inadequate treatment options that align with women's preferences, as most women with symptomatic fibroids desire minimally invasive treatments, and those younger than 40 years often prefer fertility‐preserving options [6]. While fibroids can have substantial adverse effects on pregnancy [30], successful pregnancy outcomes have been reported with TFA [23] and MIM [21, 26]. TFA provides a less invasive alternative to open and laparoscopic operations that may involve shorter surgical durations and faster recovery. For patients, the shorter recovery time may reduce the overall impact of treatment on their personal and professional lives, potentially encouraging earlier treatment‐seeking behaviour.

The clinically and statistically significant 7.5‐day shorter RTNA observed with TFA compared to MIM is a meaningful benefit that should be considered in patient counselling and decision making. For many women considering uterine fibroid treatment options, the prospect of a shorter recovery period may be highly compelling. However, these results must be interpreted within the context of individualised patient care. While TFA demonstrated several advantages in this study, MIM remains a reasonable treatment option for women with submucosal, subserous, cervical, and pedunculated fibroids, or those seeking rapid symptom relief, as soft tissue ablation typically requires 1–3 months for significant improvement [16, 18]. Ultimately, treatment selection should involve shared decision‐making between patient and provider, considering individual patient priorities, treatment risks, fibroid characteristics, and reproductive goals, as well as comprehensive counselling on the full range of available treatment options [31]. As there is no histological confirmation of fibroids with TFA, careful patient selection is necessary. In order to minimise the risk of treating rare uterine malignancy [32], the upper age limit for inclusion in this study was 50 years, and patients with unclear sonographic findings were excluded.

### 4.3 Strengths and Limitations

The key strengths of this study include the randomization design and detailed documentation of postprocedural recovery across multiple domains. Several limitations of this study also warrant mention. First, the outcomes from this single‐center study in Germany may not be generalizable to other healthcare environments. Second, this paper reports data through 7 weeks of follow‐up. Longer‐term comparative safety and efficacy of these treatments will be reported in future publications. Third, although only one serious adverse event occurred in the trial, the sample size may be insufficient to reliably detect rare complications. Fourth, although the presence of clinically significant endometriosis was an exclusion criterion, mild and moderate endometriosis was incidentally identified and concomitantly treated in 21 patients during MIM. Because these cases involved excision of superficial peritoneal endometriotic lesions, the influence of concomitant endometriosis treatment on recovery outcomes in the MIM arm is unclear. As this is a randomised controlled trial, endometriosis should theoretically affect both groups equally, although it was only diagnosed and treated in the MIM group where simultaneous laparoscopy was performed. Finally, although TFA and MIM are commonly performed as outpatient procedures, a longer hospital stay is required in our institution and in some other countries to receive full reimbursement under the national health system, which may limit comparability of discharge timing across different health systems.

## 5 Conclusions

TFA offers a faster recovery than MIM for the treatment of symptomatic uterine fibroids, with a comparable short‐term safety profile.

## Author Contributions

All authors participated in the conception, planning, and conduct of the trial. F.N., A.B., and S.Y.B. wrote the first draft of the paper. B.K., T.D., S.M., and A.R. provided critical review and revision of the paper. All authors accept responsibility for the submitted paper.

## Ethics Statement

The study protocol underwent review and approval by the ethics committee at University Hospital Tübingen (Tübingen, Germany) on 2022‐03‐07.

## Consent

All participants provided written informed consent before enrollment.

## Conflicts of Interest

The authors declare no conflicts of interest.

## Supporting information


**Figure S1:** Distribution of Fibroid Diameters Treated with Transcervical Fibroid Ablation (TFA) or Minimally Invasive Myomectomy (MIM). The mean diameter of treated fibroids was statistically higher in the TFA group (3.7 ± 2.0 vs. 3.2 ± 2.2 cm; *p* = 0.02).
**Table S1:** Characteristics of Patients Treated with Transcervical Fibroid Ablation (TFA) or Minimally Invasive Myomectomy (MIM). *Values reported as count, mean ± SD (min, max), or *n* (%) unless otherwise specified. **Reported as median (min, max). ***Median of 0.5 indicates half of participants were nulligravid and half had one or more pregnancies.
**Table S2:** Days to Return to Activities in Patients Treated with Transcervical Fibroid Ablation (TFA) or Minimally Invasive Myomectomy (MIM). *Values derived from Kaplan–Meier estimates.

## Data Availability

The authors have nothing to report.
