# Supplementary material for: Recovery After Transcervical Fibroid Ablation Versus Minimally Invasive Myomectomy for Symptomatic Uterine Fibroids: A Randomised Controlled Trial
Source: BJOG. 2025 Nov 10;133(4):618–25. doi: 10.1111/1471-0528.70081 (PMC12884227; doi:10.1111/1471-0528.70081)
Supplement: Supplementary file 1 — Figure S1: Distribution of Fibroid Diameters Treated with Transcervical Fibroid Ablation (TFA) or Minimally Invasive Myomectomy (MIM). The mean diameter of treated fibroids was statistically higher in the TFA group (3.7 ± 2.0 vs. 3.2 ± 2.2 cm; p = 0.02). Table S1: Characteristics of Patients Treated with Transcervical Fibroid Ablation (TFA) or Minimally Invasive Myomectomy (MIM). *Values reported as count, mean ± SD (min, max), or n (%) unless otherwise specified. **Reported as median (min, max). ***Median of 0.5 indicates half of participants were nulligravid and half had one or more pregnancies. Table S2: Days to Return to Activities in Patients Treated with Transcervical Fibroid Ablation (TFA) or Minimally Invasive Myomectomy (MIM). *Values derived from Kaplan–Meier estimates. [file BJO-133-618-s001.docx]

**SUPPLEMENT**

**Table S1. Characteristics of Patients Treated with Transcervical Fibroid Ablation (TFA) or Minimally Invasive Myomectomy (MIM).**

| **Variable*** | **TFA** | **MIM** |
| --- | --- | --- |
| **Patients** |  |  |
| No. patients (as randomized) | 60 | 59 |
| Age, yr | 38.6 ± 6.4 (22, 50) | 36.4 ± 5.5 (25, 48) |
| Symptoms |  |  |
| Heavy menstrual bleeding | 42 (70.0%) | 41 (69.5%) |
| Menstrual cramping/dysmenorrhea | 38 (63.3%) | 29 (49.2%) |
| Clots | 28 (46.7%) | 23 (39.0%) |
| Dyspareunia | 10 (16.7%) | 11 (18.6%) |
| Bulk symptoms | 2 (3.3%) | 1 (1.7%) |
| Gravidity** | 0.5 (0, 5) *** | 0 (0, 5) |
| Parity** | 0 (0, 4) | 0 (0, 4) |
| Miscarriage** | 0 (0, 3) | 0 (0, 2) |
| Previous fibroid procedure | 11 (18.3%) | 14 (23.7%) |
| **Fibroids** |  |  |
| No. fibroids | 119 | 108 |
| Fibroids per patient | 2.0 ± 1.4 (1, 7) | 1.8 ± 1.2 (1, 7) |
| Fibroid diameter, cm | 3.9 ± 1.9 (0.9, 8.0) | 3.9 ± 2.0 (0.3, 8.0) |
| Fibroid location relative to uterine wall |  |  |
| Transmural | 67 (56.3%) | 47 (43.5%) |
| Intramural | 29 (24.4%) | 30 (27.8%) |
| Subserosal | 11 (9.2%) | 20 (18.5%) |
| Submucosal | 12 (10.1%) | 11 (10.2%) |
| Fibroid location relative to uterine landmark |  |  |
| Body | 80 (67.2%) | 73 (67.6%) |
| Fundal | 38 (31.9%) | 31 (28.7%) |
| Lower segment | 1 (0.8%) | 4 (3.7%) |

*Values reported as count, mean ± SD (min, max), or n (%) unless otherwise specified.

**Reported as median (min, max).

***Median of 0.5 indicates half of participants were nulligravid and half had one or more pregnancies.

**Table S2. Days to Return to Activities in Patients Treated with Transcervical Fibroid Ablation (TFA) or Minimally Invasive Myomectomy (MIM).***

| **Variable** | **TFA** | | **MIM** | | **Log-rank P-value** |
| --- | --- | --- | --- | --- | --- |
|  | **Median** | **95% CI** | **Median** | **95% CI** |  |
| Normal daily activity | 5.5 | 4, 7 | 13 | 10, 17 | <0.001 |
| Normal urination | 0 | 0, 1 | 1 | 0, 2 | 0.002 |
| Eat regular diet | 1 | 0, 1 | 2 | 1, 3 | <0.001 |
| Normal sleep | 1 | 1, 2 | 3 | 2, 4 | <0.001 |
| Normal bowel movements | 3 | 2, 3 | 3 | 2, 3 | 0.25 |
| Drive | 3 | 2, 4 | 7 | 5, 10 | <0.001 |
| Perform household tasks | 4 | 4, 6 | 8 | 7, 14 | <0.001 |
| Normal recreational activity | 7 | 6, 11 | 21 | 17, 28 | <0.001 |
| Lift/carry things as normal | 7.5 | 5, 14 | 27 | 20, 35 | <0.001 |
| Return to work | 12 | 10, 14 | 18 | 16, 24 | 0.001 |
| Sexual intercouse | 15 | 11, 24 | 29 | 24, 39 | <0.001 |

*Values derived from Kaplan-Meier estimates.


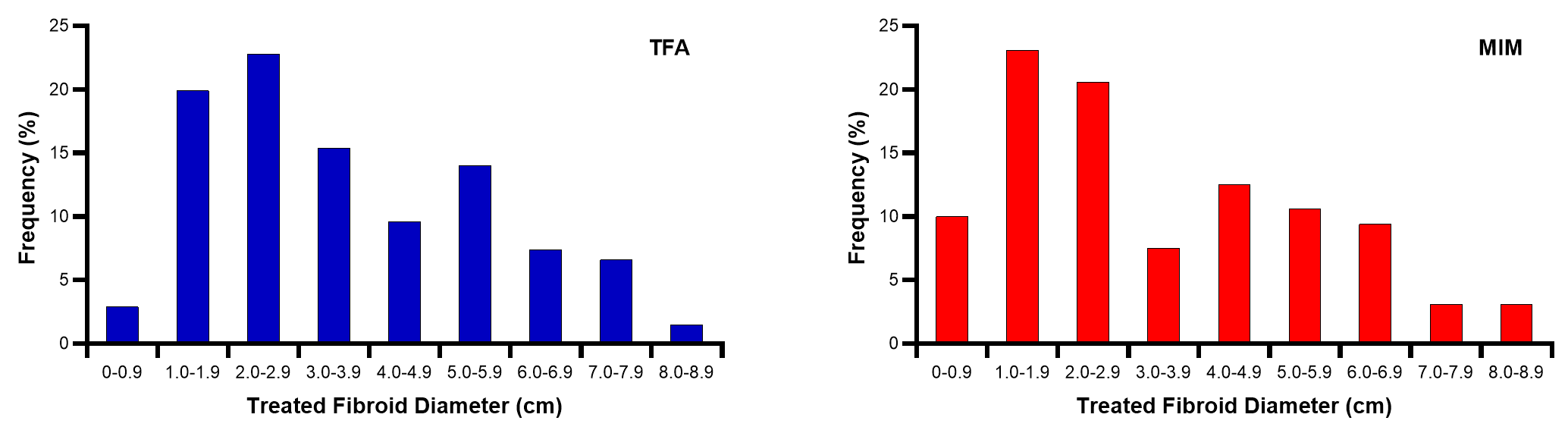


**Figure S1. Distribution of Fibroid Diameters Treated with Transcervical Fibroid Ablation (TFA) or Minimally Invasive Myomectomy (MIM).** The mean diameter of treated fibroids was statistically higher in the TFA group (3.7±2.0 vs. 3.2±2.2 cm; p=0.02).
